# A Case of a Longstanding Perianal Lesion Presenting as Advanced Squamous Cell Carcinoma of the Anus

**DOI:** 10.7759/cureus.93100

**Published:** 2025-09-24

**Authors:** Benjamin K Linkous, Nausheen Merchant, Filip Ptak, Rossana Sassarini, Gavin Harewood

**Affiliations:** 1 General Surgery, Florida State University College of Medicine, Tallahassee, USA; 2 Internal Medicine, Florida State University College of Medicine, Tallahassee, USA; 3 Neurology/Ophthalmology, Florida State University College of Medicine, Tallahassee, USA; 4 Obstetrics and Gynecology, Florida State University College of Medicine, Tallahassee, USA; 5 Gastroenterology, Cleveland Clinic Florida, Vero Beach, USA

**Keywords:** gastroenterology, hpv-negative, p16-negative, perianal lesion, quality of life, squamous cell carcinoma of the anus

## Abstract

Squamous cell carcinoma of the anus (SCCA) is a rare malignancy with rising incidence. Early-stage disease has a favorable prognosis, but advanced tumors, nodal involvement, and p16-negative status worsen outcomes. We present a 63-year-old man with psychiatric comorbidities and long-term nicotine dependence who delayed care for a four-year ulcerated perianal lesion. Imaging revealed a large invasive anal mass and bilateral inguinal lymphadenopathy (T3N1a). Biopsy confirmed poorly differentiated, HPV-negative, p16-negative SCCA. He began chemoradiotherapy with supportive care. This case underscores the impact of delayed diagnosis and tumor biology on prognosis, highlighting the need for early recognition, multidisciplinary care, and smoking cessation.

## Introduction

Squamous cell carcinoma of the anus (SCCA) is a rare but increasingly prevalent malignancy, representing approximately 1.5% of all gastrointestinal cancers [1,2]. Early-stage disease carries a favorable five-year survival rate, but delayed diagnosis is strongly associated with worse outcomes due to local invasion and nodal metastasis [2].

Epidemiologic trends highlight growing concern worldwide. In 2020, age-standardized mortality rates were highest in Central and Eastern Europe, including Slovakia, the United Kingdom, and Denmark while the lowest rates were reported in the Philippines, Mexico, and Japan [3,4]. In the United States, the incidence of squamous cell carcinoma of the anus has risen sharply, especially among elderly women and young Black men [3,4]. Over the past decades, the proportion of advanced-stage disease has tripled, with significant increases in mortality [4]. Birth cohort analyses suggest that more recent generations face a substantially higher risk for developing SCCA compared with those born in the mid-20th century [3,4].

Several risk factors contribute to the development of SCCA. Persistent infection with high-risk HPV serotypes (particularly 16 and 18) remains the most important driver [3,4]. Other factors include immunosuppression from HIV infection or post-transplant therapy, tobacco use, multiple sexual partners, receptive anal intercourse, early age of sexual debut, and a history of genital warts, all of which increase susceptibility in both men and women [3,4].

The standard treatment for locally advanced SCCA is chemoradiation following the Nigro protocol, which combines 5-fluorouracil and mitomycin-C with radiation therapy [1,5-7]. Prognosis, however, varies widely depending on tumor biology, HPV status, and timeliness of diagnosis [1,5-7]. Of particular concern is the subset of HPV-negative, p16-negative tumors, which are more aggressive and associated with poorer outcomes [8]. As HPV vaccination reduces the incidence of HPV-related tumors, the relative burden of HPV-independent cancers is expected to increase [8].

This case report describes a 63-year-old man with a multi-year history of an ulcerative perianal lesion, ultimately diagnosed as HPV-negative, p16-negative, poorly differentiated SCCA. His delayed diagnosis and extensive tumor burden underscore the complexity and morbidity of advanced anal cancer management, while highlighting the importance of recognizing and studying this aggressive, non-HPV-driven subset.

## Case presentation

**Figure 1:**
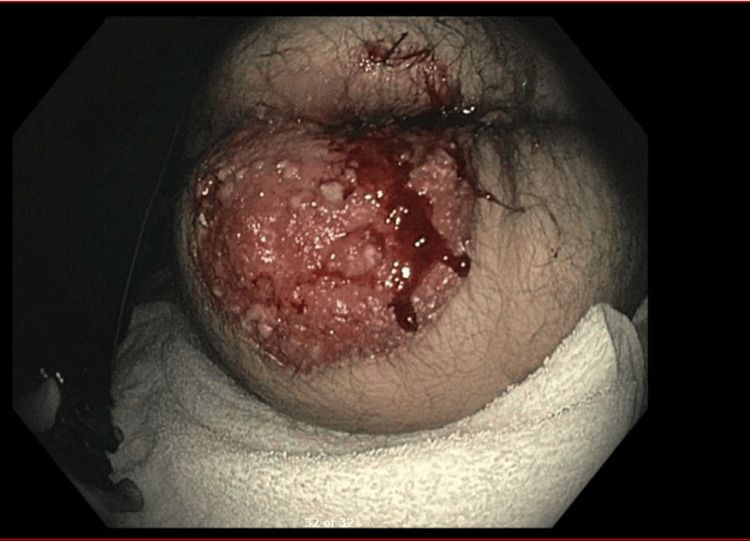
Endoscopic image of the perianal lesion Endoscopic image depicting a large, ulcerated perianal cutaneous lesion predominantly on the left buttock with irregular, friable borders and diffuse erythema. The surface is disrupted by scattered white-to-yellow keratinized plaques and areas of necrosis, with evidence of active bleeding.

A 63-year-old man with a history of anxiety, depression, nicotine dependence (20 pack years), and chronic urinary retention presented to his primary care physician with a four-year history of a progressively enlarging, ulcerated lesion on the bilateral medial buttocks extending out onto the left buttock. The lesion initially developed following an episode of constipation and hemorrhoids, which he self-managed without medical evaluation. Over time, the lesion expanded significantly, began draining serous fluid, and caused severe pain, interfering with daily activities such as sitting and sleeping. On physical examination, a 4 x 5 cm irregular, ulcerated mass was noted in the perianal region (Figure 1). Given the concerning features, the patient was referred to gastroenterology, oncology, and colorectal surgery for further evaluation. 

**Figure 2:**
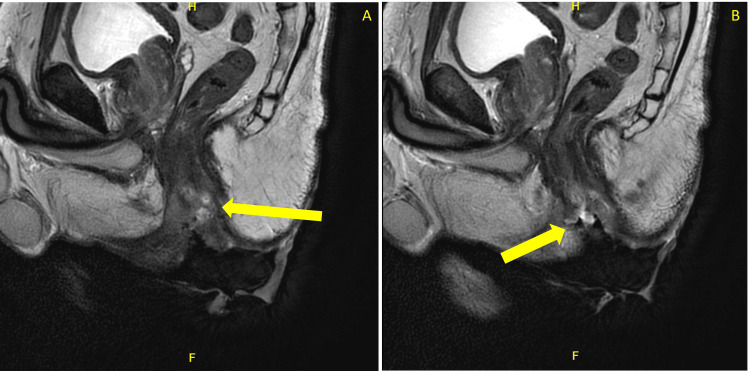
Sagittal T2-weighted pelvic MRI with contrast Sagittal T2-weighted pelvic MRI showing a heterogeneously enhancing anal canal lesion with sphincter involvement (yellow arrows). The mass distorts normal anorectal anatomy (Panel A) and extends toward surrounding soft tissues (Panel B). The letters “H” and “F” assist with image orientation and indicate head and feet, respectively.

**Figure 3:**
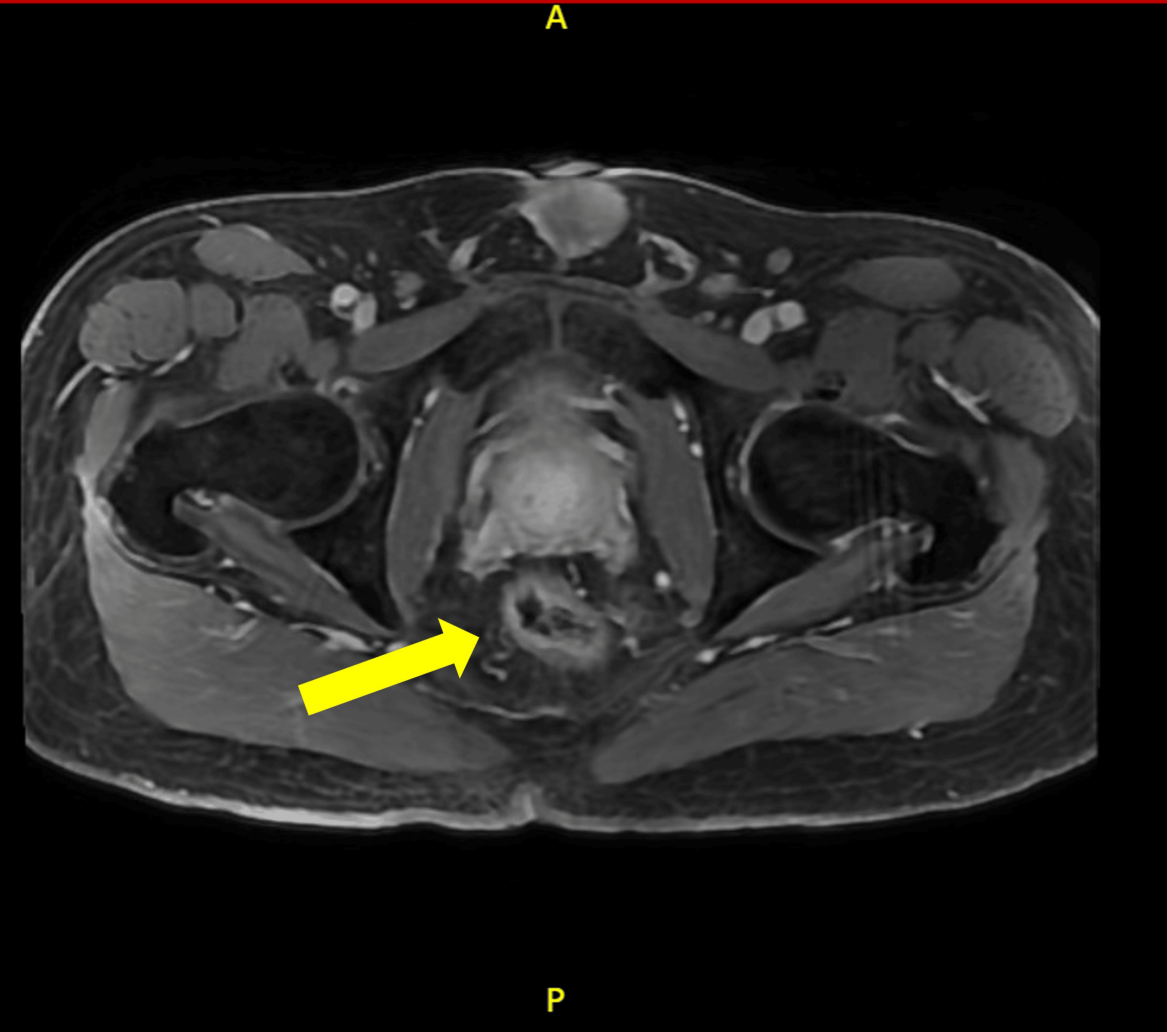
Axial T2-weighted pelvic MRI with contrast Axial T2-weighted pelvic MRI showing an intermediate signal mass involving the left aspect of the mid to lower anus (yellow arrow). The letters “A” and “P” assist with image orientation and indicate anterior and posterior, respectively.

**Figure 4:**
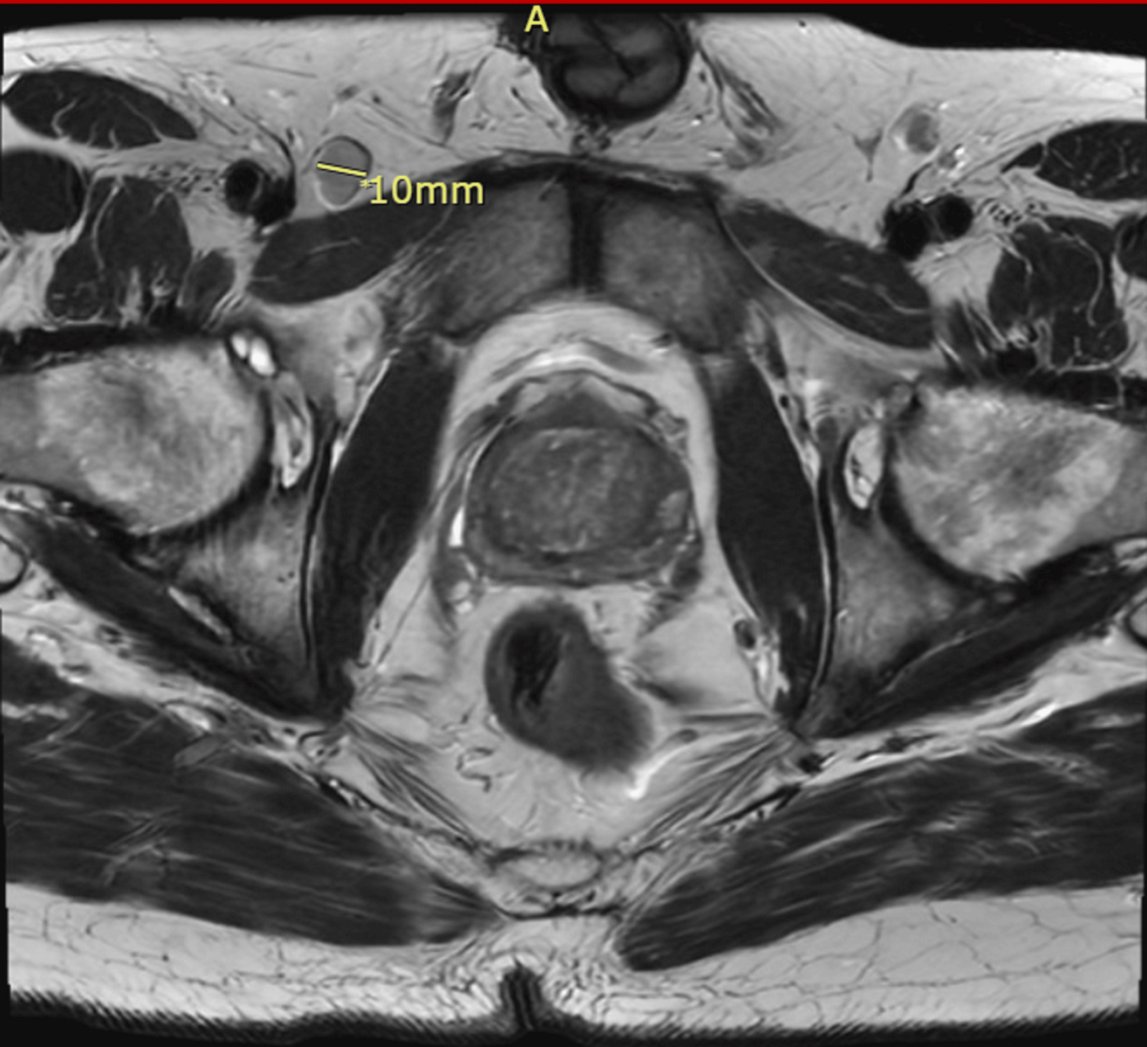
Axial T2-weighted pelvic MRI Axial T2-weighted pelvic MRI demonstrating a 10 mm right inguinal lymph node (label demonstrates short axis diameter), demonstrating concern for regional nodal metastasis in the context of an external perianal lesion. The letter “A” assists with image orientation and indicates the anterior of the patient.

Gastroenterology performed a colonoscopy due to initial concerns of the lesion being pyoderma gangrenosum secondary to ulcerative colitis. Rather, the colonoscopy revealed multiple tubular adenomas in the cecum, ascending colon, and sigmoid colon. These were resected, but the perianal lesion was not biopsied during the procedure. The gastroenterologist documented a large, ulcerated mass extending toward the anus and recommended cross-sectional imaging and tissue diagnosis. There was no extension of the lesion into the distal rectum. An MRI of the pelvis was obtained, revealing a T2 intermediate signal mass involving the mid to lower anus, extending into both the internal and external anal sphincters, the perineum, and the skin of the bilateral buttocks (Figures 2, 3). The primary lesion measured approximately 6.7 x 1.5 cm, with associated skin involvement measuring up to 12 x 12 cm. Bilateral inguinal lymphadenopathy was present, with the largest node measuring 1 cm in short-axis diameter, consistent with N1a staging (Figure 4). 

The patient was then evaluated by colorectal surgery, where multiple 3 mm punch biopsies were obtained. Pathology confirmed poorly differentiated, nonkeratinizing, p16-negative squamous cell carcinoma, with positive CK5/6 and p63 staining. The lesion extended to the biopsy margins, indicating incomplete excision. HPV testing was performed, yielding a negative result. Based on clinical and imaging findings, the patient was diagnosed with T3 N1a SCCA with extensive local and nodal involvement. 

He was referred to oncology, and a PET/CT scan of the chest, abdomen, and pelvis was performed. While no distant metastases were identified, the scan revealed incidental findings of mild prostatomegaly and circumferential bladder wall thickening. Two months after initial presentation, a 7 French low-profile Infuse-a-Port was placed utilizing the Seldinger technique to access the left subclavian vein under fluoroscopic guidance for future chemotherapy administration (Nigro protocol). The patient was provided with chemotherapy education and ondansetron for possible nausea. Furthermore, the patient will continue to work with pain management with the current treatment plan utilizing morphine ER/IR. 

## Discussion

This case illustrates the clinical and prognostic complexity associated with advanced SCCA, particularly in the setting of delayed diagnosis and aggressive tumor biology. While early-stage SCCA is highly treatable with five-year survival rates exceeding 75%, outcomes worsen significantly in patients with bulky tumors, nodal involvement, or p16-negative status [6-10]. 

The patient’s prolonged symptom duration allowed for substantial local progression, including perineal invasion, sphincter involvement, and ulceration of the buttocks. These features reflect common pitfalls in early detection, as symptoms such as pain, irritation, or presumed hemorrhoids are frequently misattributed and undertreated [11,12]. By the time of diagnosis, the lesion exceeded 6 cm and exhibited bilateral inguinal nodal involvement, portending a poor prognosis with an estimated five-year survival of around 50% [2]. Staging is a critical prognostic tool in SCCA, and according to version 9 of the American Joint Committee on Cancer (AJCC) staging systems for anal cancer, tumor size and nodal status remain central to risk stratification [13,14]. Table 1 outlines the AJCC staging system and demonstrates how this patient met criteria for T3N1aM0 disease, corresponding to Stage IIIA [13,14].

**Table 1:** AJCC cancer staging system version 9: anus This staging system was developed and validated by the American Joint Committee on Cancer (AJCC) to restore hierarchical prognostic order and improve survival discrimination, as supported by population-level analyses and survival outcome data. Source: Adapted from data in [13,14]

T Category	N Category	M Category	Criteria Summary	Stage Group
T1	N0	M0	Tumor ≤ 2 cm in greatest dimension; no regional node involvement; no distant metastasis	I
T2	N0	M0	Tumor > 2 cm but ≤ 5 cm; no regional node involvement; no distant metastasis	IIA
T1–T2	N1 (N1a/N1b/N1c)	M0	Tumor ≤ 5 cm; lymph node involvement (1a - inguinal, mesorectal, rectal, iliac, or obturator node(s); 1b - external iliac node(s); 1c - external iliac node(s) with any 1a nodes); no distant metastasis	IIB
T3	N0–N1	M0	Tumor > 5 cm; may or may not involve regional nodes; no distant metastasis	IIIA
T4	N0	M0	Tumor of any size invading adjacent organs (i.e., vagina, urethra, bladder); no nodal involvement; no distant metastasis	IIIB
T4	N1 (N1a/N1b/N1c)	M0	Tumor of any size invading adjacent organs; with lymph node involvement (1a - inguinal, mesorectal, rectal, iliac, or obturator node(s); 1b - external iliac node(s); 1c - external iliac node(s) with any 1a nodes); no distant metastasis	IIIC
Any T	Any N	cM1/pM1	Any tumor size and nodal status with distant metastasis (clinical or pathologic confirmation)	IV

Most SCCAs are HPV-positive and p16-overexpressing, which confers a favorable response to chemoradiotherapy [15]. However, approximately 9% are HPV-negative and p16-negative, a subset associated with poor therapy response rates and high recurrence risk [15]. Furthermore, p16-negative tumors frequently harbor p53 mutations, contributing to radioresistance and poor locoregional control (as low as 15%) [5-7,15]. As such, p16 testing should be considered early in the diagnostic process, not only as a prognostic biomarker but also to inform surveillance intensity and discussions regarding therapeutic expectations.

Immunohistochemistry markers such as Ki-67, p16, and HPV-E4 help characterize anal intraepithelial neoplasia and its progression risk, with Ki-67 reflecting proliferative activity that increases with higher-grade lesions [16]. CK7/20 profiling can assist in distinguishing SCCA from other anogenital or colorectal tumors, as SCCA typically shows a CK7+/CK20- pattern, supporting HPV-driven squamous differentiation [16]. While these markers provide diagnostic and biological insight, DNA methylation remains more reliable than immunohistochemistry alone for predicting progression and prognosis in SCCA [16].

This patient’s tumor burden translated into persistent pain, impaired mobility, and sleep disruption, all of which impacted his daily functioning. Chronic wound drainage, bowel dysfunction, and sexual impairment are additional sequelae of disease progression, with profound emotional and psychosocial consequences [17,18]. If salvage surgery, such as abdominoperineal resection, becomes necessary, the risks for colostomy dependence, delayed perineal healing, and substantial lifestyle disruption must be carefully weighed against potential benefits [19]. These realities highlight the critical need for multidisciplinary care, including preoperative counseling and robust psychosocial support.

Compounding these clinical challenges was the patient’s long-standing tobacco use, an established risk factor for anal cancer that is independently associated with higher recurrence rates and reduced treatment response [17-20]. Integrating smoking cessation support into care planning is essential, as even cessation at diagnosis has been associated with improved outcomes [17-20].

## Conclusions

This case highlights the importance of early recognition of SCCA symptoms, particularly when lesions are persistent, painful, or ulcerated. Accurate staging, awareness of tumor biology and timely intervention are critical to improving outcomes. Moreover, care must extend beyond oncologic treatment to include multidisciplinary efforts addressing pain, function, and psychosocial well-being. This comprehensive approach is especially important in patients with HPV-independent disease, where aggressive tumor behavior and treatment resistance demand individualized, coordinated care. Public education, smoking cessation, and provider awareness are key to promoting earlier detection and reducing morbidity.
